# Possible molecular exploration of herbal pair Haizao-Kunbu in the treatment of Graves’ disease by network pharmacology, molecular docking, and molecular dynamic analysis

**DOI:** 10.3389/fendo.2023.1236549

**Published:** 2023-10-04

**Authors:** Mengfei Yang, Yiwen Lai, Di Gan, Qingyang Liu, Yingna Wang, Xinyong He, Yi An, Tianshu Gao

**Affiliations:** ^1^ Graduate School, Liaoning University of Traditional Chinese Medicine, Shenyang, Liaoning, China; ^2^ Department of Endocrinology, The Affiliated Hospital of Liaoning University of Traditional Chinese Medicine, Shenyang, Liaoning, China; ^3^ Insititute of Laboratory Medicine, Liaoning University of Traditional Chinese Medicine, Shenyang, Liaoning, China; ^4^ Department of Obstetrics, The People’s Hospital of Liaoning, Shenyang, Liaoning, China

**Keywords:** Haizao-Kunbu, Graves’ disease, network pharmacology, molecular docking, molecular dynamic analysis statements and declarations

## Abstract

**Objective:**

To promote the development and therapeutic application of new medications, it is crucial to conduct a thorough investigation into the mechanism by which the traditional Chinese herb pair of Haizao-Kunbu (HK) treats Graves’ disease (GD).

**Materials and methods:**

Chemical ingredients of HK, putative target genes, and GD-associated genes were retrieved from online public databases. Using Cytoscape 3.9.1, a compound-gene target network was established to explore the association between prosperous ingredients and targets. STRING, Gene Ontology, and Kyoto Encyclopedia of Genes and Genomes pathway analyses visualized core targets and disease pathways. Additionally, we conducted a refined analysis of the binding interactions between active ingredients and their respective targets. To visualize these findings, we employed precise molecular docking techniques. Furthermore, we carried out molecular dynamics simulations to gain insights into the formation of more tightly bound complexes.

**Results:**

We found that there were nine key active ingredients in HK, which mainly acted on 21 targets. These targets primarily regulated several biological processes such as cell population proliferation, protein phosphorylation, and regulation of kinase activity, and acted on PI3K-AKT and MAPK pathways to treat GD. Analysis of the molecular interaction simulation under computer technology revealed that the key targets exhibited strong binding activity to active ingredients, and Fucosterol-AKT1 and Isofucosterol-AKT1 complexes were highly stable in humans.

**Conclusion:**

This study demonstrates that HK exerts therapeutic effects on GD in a multi-component, multi-target, and multi-pathway manner by regulating cell proliferation, differentiation, inflammation, and immunomodulatory-related targets. This study provides a theoretical foundation for further investigation into GD.

## Introduction

Graves’ Disease (GD) is an autoimmune illness characterized by an enlarged and hyperactive thyroid gland (Graves’ hyperthyroidism), ocular abnormalities (Graves’ orbitopathy), and regional dermopathy (pretibial myxoedema). After two decades of Universal Salt Iodization (USI), GD prevalence in China has gradually decreased, with the latest epidemiological survey results at 0.53% (1). However, this implies that about seven million people still have GD. The development of GD in patients is initiated with a genetic predisposition, further influenced by some environmental factors. The complex interplay manifests through thyroid follicular cell proliferation and hypertrophy and abnormal immune cell proliferation and differentiation, which is essentially the imbalance of cell value-added apoptosis driving GD hyperthyroidism occurrence. Meanwhile, recent studies have shown that elevated levels of Th1 chemokines, such as CXCL10, are found in the serum of patients with relapsed and newly diagnosed hyperthyroidism. Th1 immune response predominates in the immunopathogenesis of GD, causing the emergence and persistence of autoimmune inflammation in the thyroid gland. The onset and treatment of GD are also closely related to cytokines such as TNF-α and IL-6 (2, 3).

Controlling hyperthyroidism by restoring standard thyroid hormone concentrations is the principal objective of GD treatment. Some therapeutic options for Graves’ hyperthyroidism patients include Antithyroid Drugs (ATDs), radioiodine, and surgery. However, these treatment options have some drawbacks. Antithyroid medicines produce granulocyte count and liver enzyme abnormalities, and recurrence occurs in > 50% of cases after terminating ATDs (4). On the other hand, radioiodine or surgery may trigger hypothyroidism, necessitating lifetime levothyroxine implementation as well as clinical and laboratory supervision (5). Furthermore, treating special populations of GD patients, such as pregnant and lactating women or patients with malignant tumors, is a difficulty that cannot be overlooked.

There are records of Yingbing (goiter, 瘿病) in Traditional Chinese Medicine (TCM). The records are derived from a Chinese medicine text, *Zhu Bing Yuan Hou Lun*, which focusses on diagnosis and management of a class of diseases manifested as goiter, and GD is one of them. According to *Waike Zhengzong*, Yingbing is “not swelling of yin and yang, but stasis of blood, turbidity, and phlegm in the five organs.” Consequently, doctors mostly use Chinese medications that reduce hardness and disperse stagnation for goiter treatment. From inception to the present time, the herb pair Haizao-Kunbu (HK), first appearing in *Zhou Hou Bei Ji Fang*, has been a common and effective combination therapy for goiter. According to *Shennong′s Classic of the Materia Medica*, Haizao (Sargassum) can “master goiter, tumor qi, and the nucleus of the neck.” Additionally, *Mingyi Bielu* reported that Kunbu (Laminaria japonica) could “treat twelve kinds of edema and goiter tumor coalescing gas.” Some TCM formulas containing HK for goiter treatment include the Sihai Shuyu pill from *Yangyi Daquan*, the Haizao Yuhu decoction from *Waike Zhengzong*, and the Huaying Micro pill from *Rumen Shiqin*. Previous pharmacological research has demonstrated that besides reducing autoimmune antibody levels in rats with thyroid disease (6–8), Haizao has properties that can suppress cell proliferation and apoptosis induction (9). Kunbu, on the other hand, has enormous clinical benefits, including anti-cell proliferation, angiogenesis inhibition, apoptosis blocking, anti-inflammatory, and antioxidant properties (10, 11). Furthermore, Haizao and Kunbu are considered natural medicines with minimal side effects, given their role in protecting liver and kidney function (12, 13). However, the precise mechanism of HK in GD treatment remains unclear. For the first time, this study will elucidate the molecular mechanisms underlying this well-known combination treatment for GD.

In TCM, there is a focus on adjusting the integrity of the human body based on the balance-regulation theory. However, due to the complex character of TCM, research on its pharmacological mechanism is challenging. Network pharmacology highlights the multi-directional signaling pathway modulation, leading to enhanced therapeutic drug benefits and decreased toxic and adverse effects, increasing the potential efficacy of new pharmaceutical clinical trials, and lowering drug discovery costs. Molecular docking, on the other hand, is a simulated testing approach that models the geometry and interactions between molecules and proteins, allowing for investigations of molecular behavior at target protein binding sites (14). Finally, Molecular Dynamics (MD) modeling is a sophisticated *in silico* tool for exploring biological processes and molecular frameworks underlying linkages among macromolecules and ligands (15).

**Figure 1:**
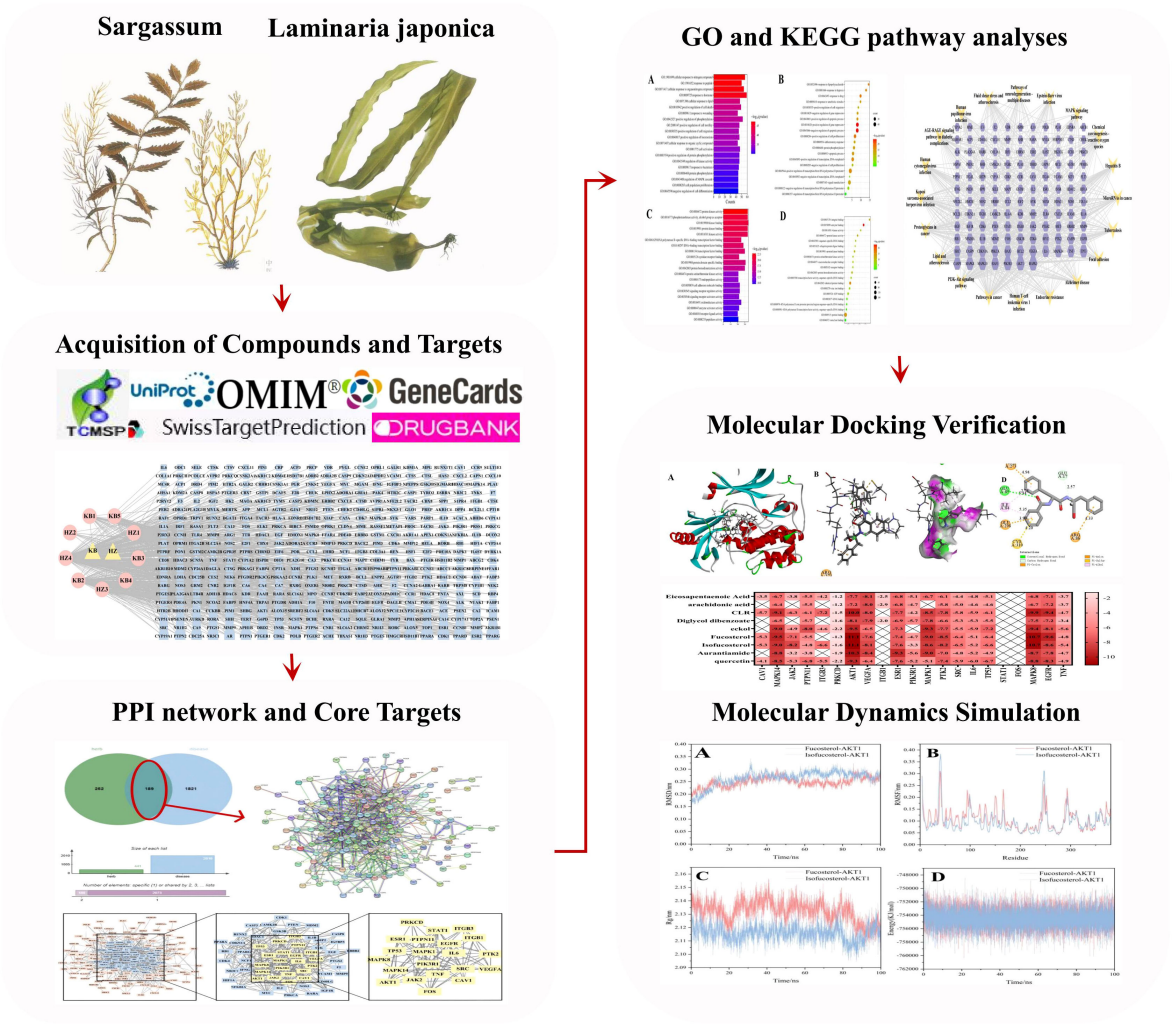
The overall workflow of the study.

Here, we used network pharmacological analysis, molecular docking, and MD simulation technology to create a “compound-target-pathway” network. The HK chemical compounds were collected and screened for Oral Bioavailability (OB) and Drug-Likeness (DL). Subsequently, public databases were reviewed for target and GD-related genes. To predict core compositions and targets that probably participated in GD treatment, we created a network associating medicinal ingredients with target genes in HK. Additionally, Protein-Protein Interaction (PPI), Gene Ontology (GO), and Kyoto Encyclopedia of Genes and Genomes (KEGG) analyses were utilized to identify potential targets and pathways. The molecular docking and MD modeling techniques provide a foundation for further research on the HK molecular processes in GD treatment. **Figure 1** depicts the study design.

## Methods

### Screening of active compounds

Potential compounds in Sargassum and Laminaria japonica were detected after searching the Traditional Chinese Medicine Systems Pharmacology (TCMSP) Database (http://tcmspw.com/) (16) and reassessing pertinent literature. The screening criteria included OB ≥ 30% and DL ≥ 0.18. The SwissADME online platform (www.swissadme.ch) was used to screen all ingredients (17).

### Prediction of targets for the HK herb pair

The TCMSP database and Swiss Target Prediction platform (www.swisstargetprediction.ch) (18) were used to predict the gene targets, and the corresponding Sargassum and Laminaria japonica targets were collected. The PubChem Compound Identifier (CID) number of each active ingredient was obtained from TCMSP, and potential targets were updated via Swiss Target Prediction with reference to the SMILE codes and 2D images of compounds obtained from PubChem (https://pubchem.ncbi.nlm.nih.gov) (19) in SDF format. After officially annotating the potential targets on the UniProt database (https://www.uniprot.org) (20), we eventually built the “herb-compound-target” network using Cytoscape 3.9.1 software (21).

### Acquisition of GD targets

To identify the target genes appertained to GD, we searched the OMIM (https://www.omim.org) (22), GeneCards (https://www.genecards.org) (23), Drugbank (https://go.drugbank.com) (24), and Disgenet (https://www.disgenet.org) (25) databases using “Graves’ disease” as the passphrase. The online Venn mapping website (26) was eventually used to map the common HK and GD targets (26). Obvolute proteins were then regarded as immanent therapeutic targets for GD intervention.

### Protein-protein interaction network building and core target identification

After importing the common targets into STRING, we built a PPI network to further explore the role of crucial targets. In our investigation, we selected interactions with the highest confidence score (≥ 0.900) for ‘Homo sapiens’, among which we focused on the least demanding interrelation score. The unconnected nodes were concealed and the connection network was illustrated using Cytoscape 3.9.1. Additionally, the CytoNCA (Cytoscape software add-in) (27) was installed to examine the topological characteristics of the targets. The PPI network was used to identify key targets based on Degree value (Degree), Closeness Centrality (CC), and Betweenness Centrality (BC).

### Gene ontology and Kyoto encyclopedia of genes and genomes pathway enrichment analyses

To further clarify the HK gene occupation and the role of intrinsic signaling pathways in GD, Metascape (https://metascape.org) (28) and David (https://david.ncifcrf.gov) (29) were used to assess GO and KEGG pathways of 189 target genes. The Bioinformatics platform (https://www.bioinformatics.com.cn) was used to plot the bar and bubble charts with color gradients for data analysis and visualization. The FDR error control technique was used to establish whether biological processes differed significantly. After correcting the *p*-value, a significance threshold of *p <*0.05 was used.

### The molecular docking process between active ingredients and key targets

Here, we used computer-assisted technology to further confirm the intensity of the interaction between the targets and the core compound. Molecular forms of key protein targets and mol2 files of the structures of ingredients were obtained from the Protein Data Bank (PDB) (https://www.rcsb.org) (30) and TCMSP databases, respectively. The PyMOL software was used to eliminate the initial ligands and water molecules of proteins (31). The AutoDock 4.2 program (32) was used to phosphorylate and store the receptor in the PDBQT file. AutoDock Vina (33) was utilized to dock and determine a superior model. Using binding free energy, all molecules and disease targets were ranked based on their interaction strength after docking simulations. The docking was considered valid when the binding free energy was < 5.0 kcal/mol. Finally, each target’s highest binding energy component was visualized using Discovery Studio 2019 Client, and GraphPad Prism 8.0.2 (GraphPad, CA, USA) was used to draw the binding energy heatmap.

### Molecular dynamics simulation

Ligand-receptor docked complex MD simulation was performed using GROMACS (version 2021.2) (34). Whereas the ligand topology file was produced by the ACPYPE script using the AMBER forefield, the protein topology file was created using the AMBER99SB-ILDN force field. For MD simulation, TIP3 water molecules were applied in a triclinic box, and periodic boundary conditions were employed. The system was neutralized using NaCl counter ions. Prior to MD simulation, the complex was reduced for 1000 steps and equilibrated by running NVT and NPT for 100 ps. The MD simulation for each system was run for 100 ns under periodic boundaries at 310 K and 1.0 bar of pressure. Finally, the free binding energy of a simulated target-ligand complex was computed using the gmx_mmpbsa tool from GROMACS.

## Results

### Screening of ingredients and selection of gene targets

**Figure 2:**
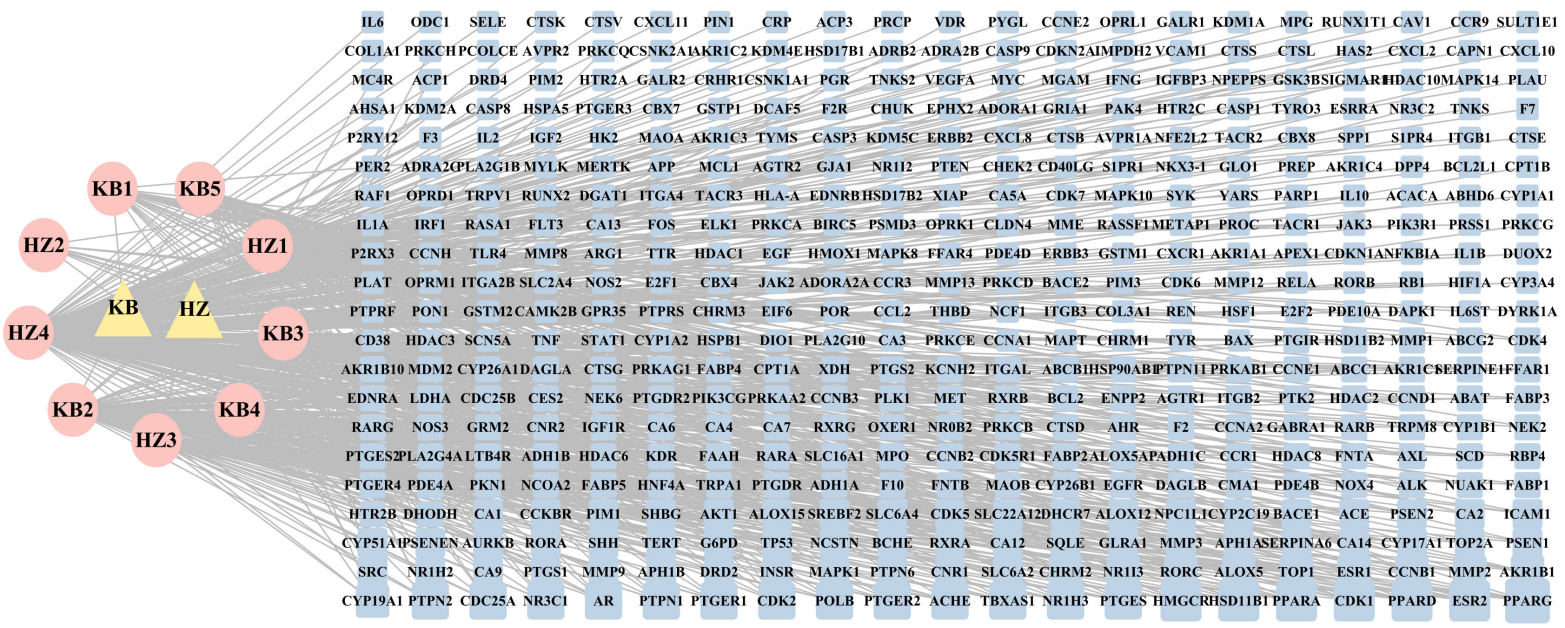
The ‘Herb-Chemical composition-Target’ network. The pink ellipse represents the medicinal herb of HK; the pink eclipse indicates the key components screened from HK. The blue round rectangle represents the key target points, and the edge connects the target to the active ingredient. In the network, a higher degree value is represented by a greater number of links and larger nodes, indicating that the active ingredient or target holds greater significance.

We selected 11 components by searching the literature and online platforms and qualified nine ingredients through the screening threshold. **Table 1** details the active ingredients. We identified 441 compound targets after deleting the duplicate and unreviewed genes. The HK “Chemical composition-target” network showing the connection between the nine components and 441 target genes was built using Cytoscape 3.9.1. The general characteristic of the network analysis was estimated to be 452 nodes and 751 edges (**Figure 2**).

**Table 1:** The details of the active compounds in HK herb pair.

Serial number	The scientific name of Chinese medicine	Family	Mol ID	Molecule Name	Molecule Formula	MW	AlogP	Hdon	Hacc	OB (%)	DL
KB1	Thalluslaminariae	Laminariaceae	MOL010616	eckol	C18H12O9	372.3	3.08	6	9	87.06	0.63
KB2	Thalluslaminariae	Laminariaceae	MOL010617	Eicosapentaenoic Acid	C20H30O2	302.5	5.97	1	2	45.66	0.21
KB3	Thalluslaminariae	Laminariaceae	MOL001439	arachidonic acid	C20H32O2	304.52	6.41	1	2	45.57	0.2
KB4	Thalluslaminariae	Laminariaceae	MOL000953	CLR	C27H46O	386.73	7.38	1	1	37.87	0.68
KB5	Thalluslaminariae	Laminariaceae	MOL009622	Fucosterol	C29H48O	412.77	7.83	1	1	43.78	0.76
HZ1	Sargassum	Sargassaceae	MOL010578	Aurantiamide	C25H26N2O3	402.53	3.64	3	5	45.76	0.43
HZ2	Sargassum	Sargassaceae	MOL010580	Diglycol dibenzoate	C18H18O5	314.36	3.06	0	5	59.22	0.27
HZ3	Sargassum	Sargassaceae	MOL005440	Isofucosterol	C29H48O	412.77	7.83	1	1	43.78	0.76
HZ4	Sargassum	Sargassaceae	MOL000098	quercetin	C15H10O7	302.25	1.5	5	7	46.43	0.28

### GD targets searching

**Figure 3:**
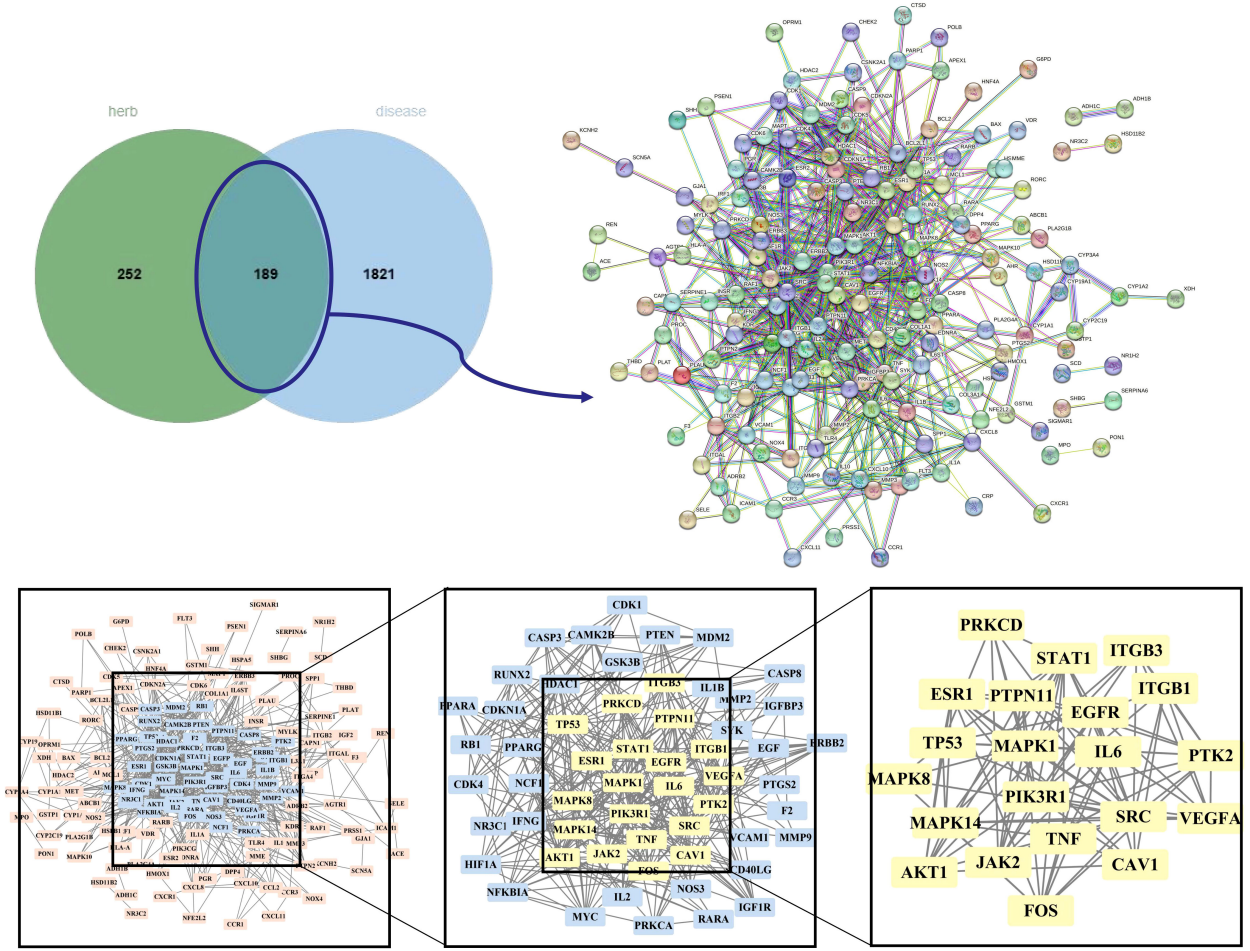
Venn plot, PPI network diagram, and screening topology of core targets of HK treatment for GD.

The OMIM, DrugBank, GeneCards, and Disgenet databases yielded 570, 12, 2470, and 585 GD targets, respectively. After collecting all the genes and removing duplicate data, 2,010 targets remained for further research (**Supplementary Table S1**). Finally, the Venn diagram displayed 189 genes as the herb pair’s implicit aim for GD therapies (**Figure 3**).

### PPI analysis

We imported 189 potential targets into STRING and obtained 165 potential targets through the screening threshold. Subsequently, we created a PPI network (165 nodes and 771 edges) after importing target genes into Cytoscape 3.9.1 software (**Figure 3**). Increasing quantified values were associated with the improved significance of the node. The key target topological analysis was based on Degree, CC, and BC > one-fold of the median. We first sorted 21 key gene targets using the Degree > 7, CC > 0.393, and BC > 0.003 criteria and then sorted 19 key gene targets using the Degree > 12, CC > 0.433, and BC > 0.013 criteria (**Supplementary Table S2**).

### GO terms and KEGG pathways

**Figure 4:**
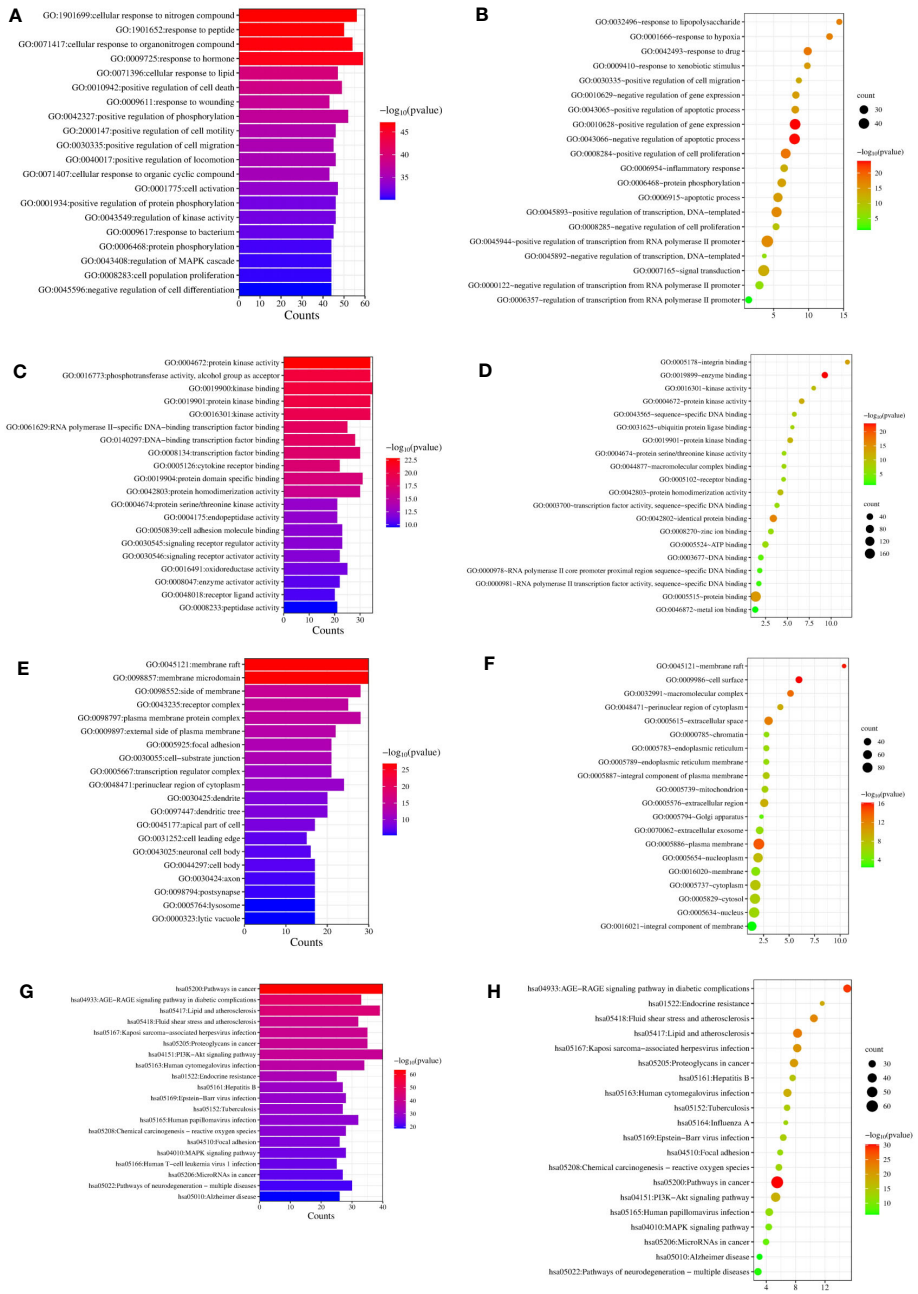
GO and KEGG analysis of HK in the treatment of GD. **(A, C, E, G)** Biological process, molecular function, cell component, and KEGG enrichment pathways from Metascape database. **(B, D, F, H)** Biological process, molecular function, cell component, and KEGG enrichment pathways from David database.

We completed GO and KEGG enrichment studies to further demonstrate the proposed targets’ organic functions and prospective mechanisms. The analyses involved 189 HK potential target genes underlying GD. The results were obtained after analysis of the GO and KEGG enrichment results analyzed by Metascape and David databases (**Figure 4**). We discovered that the common GO biological processes in the two databases were protein phosphorylation (GO: 0006468) and positive cell migration regulation (GO: 0030335), implying that phosphorylation and cell migration may be significantly involved in GD treatment by HK. Additionally, we discovered that protein homodimerization activity (GO: 0042803), protein kinase binding (GO: 0019901), protein kinase activity (GO: 0004672), kinase activity (GO: 0016301), and protein serine/threonine kinase activity (GO: 0004674) were simultaneous entries among the GO molecular functions. This finding indicates that protein kinase activity was crucial throughout the GD treatment process. Furthermore, terms such as cytoplasm perinuclear region (GO: 0048471) and membrane raft (GO: 0045121) were found to be crucial cellular components depending on the cellular composition. Cell membranes and cytoplasm are vital organelles that regulate cell signaling.

**Figure 5:**
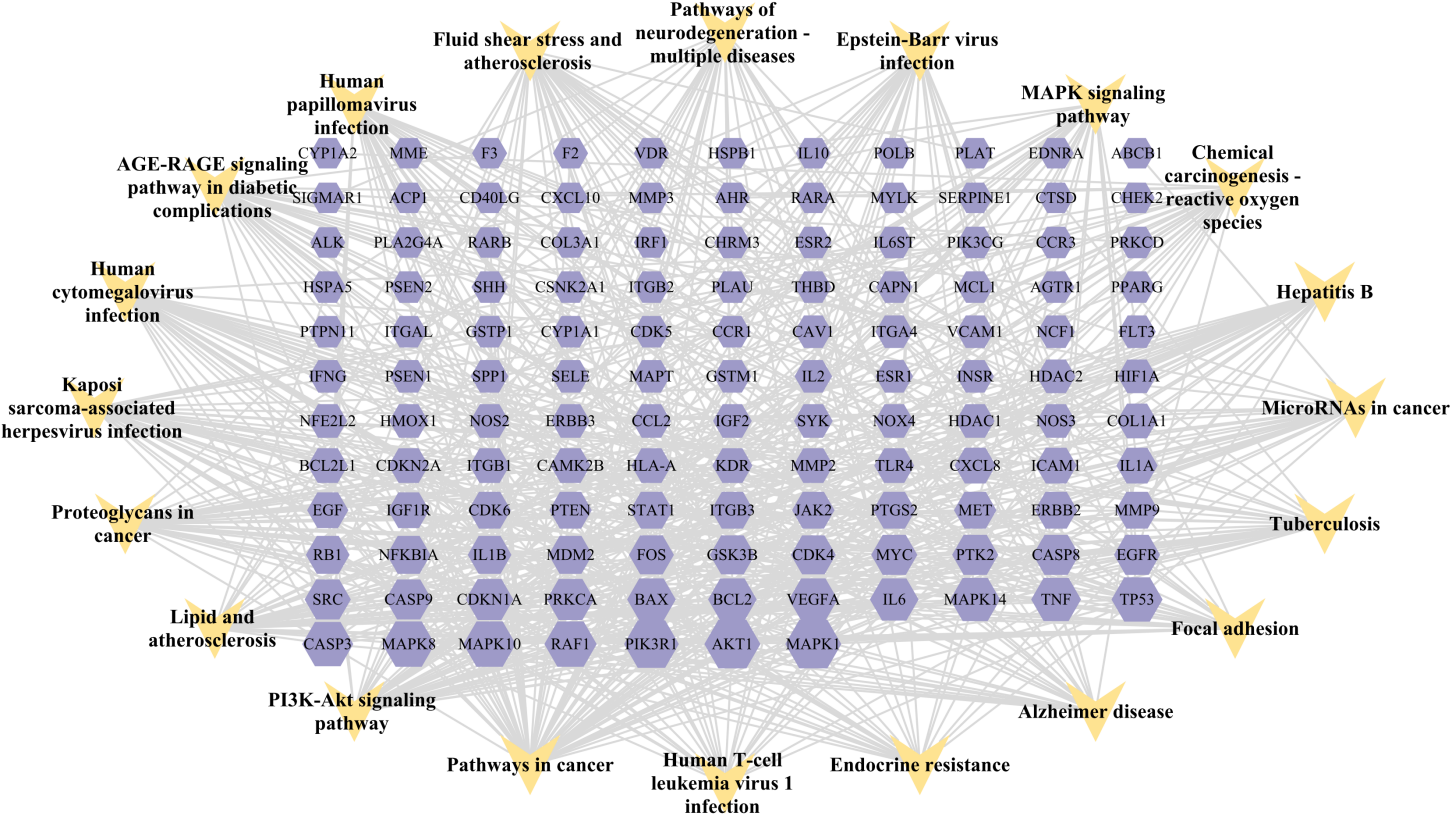
**“**Target gene-KEGG pathways” network: genes are indicated by the purple hexagon and pathways are presented as yellow V-shapes.

A KEGG pathway enrichment assessment was performed to further investigate the possible functions of the targets (P<0.05). The following 19 pathways appeared simultaneously in the top 20 critical pathways analyzed by the two databases: Cancer Pathways (hsa05200), the PI3K-AKT Signaling Pathway (hsa04151), Lipid and Atherosclerosis (hsa05417), Proteoglycans in Cancer (hsa05205), Human Cytomegalovirus (HCMV) Infection (hsa05163), Kaposi Sarcoma-associated Herpesvirus (KSHV) Infection (hsa05167), Fluid Shear Stress and Atherosclerosis (hsa05418), the AGE-RAGE Signaling Pathway in Diabetic Complications (hsa04933), Human Papillomavirus (HPV) Infection (hsa05165), Neurodegeneration-multiple Disease Pathways (hsa05022), the MAPK Signaling Pathway (hsa04010), Hepatitis B (hsa05161), Tuberculosis (hsa05152), Epstein-Barr Virus (EBV) Infection (hsa05169), Chemical Carcinogenesis-Reactive Oxygen Species (hsa05208), MicroRNAs in Cancer (hsa05206), Focal Adhesion (hsa04510), Alzheimer’s Disease (hsa05010), and Endocrine Resistance (hsa01522). Based on this outcome, we finally constructed a “KEGG pathway-gene target” network (**Figure 5**). Detailed information is provided in **Supplementary Table S3**.

### Molecular docking verification

**Figure 6:**
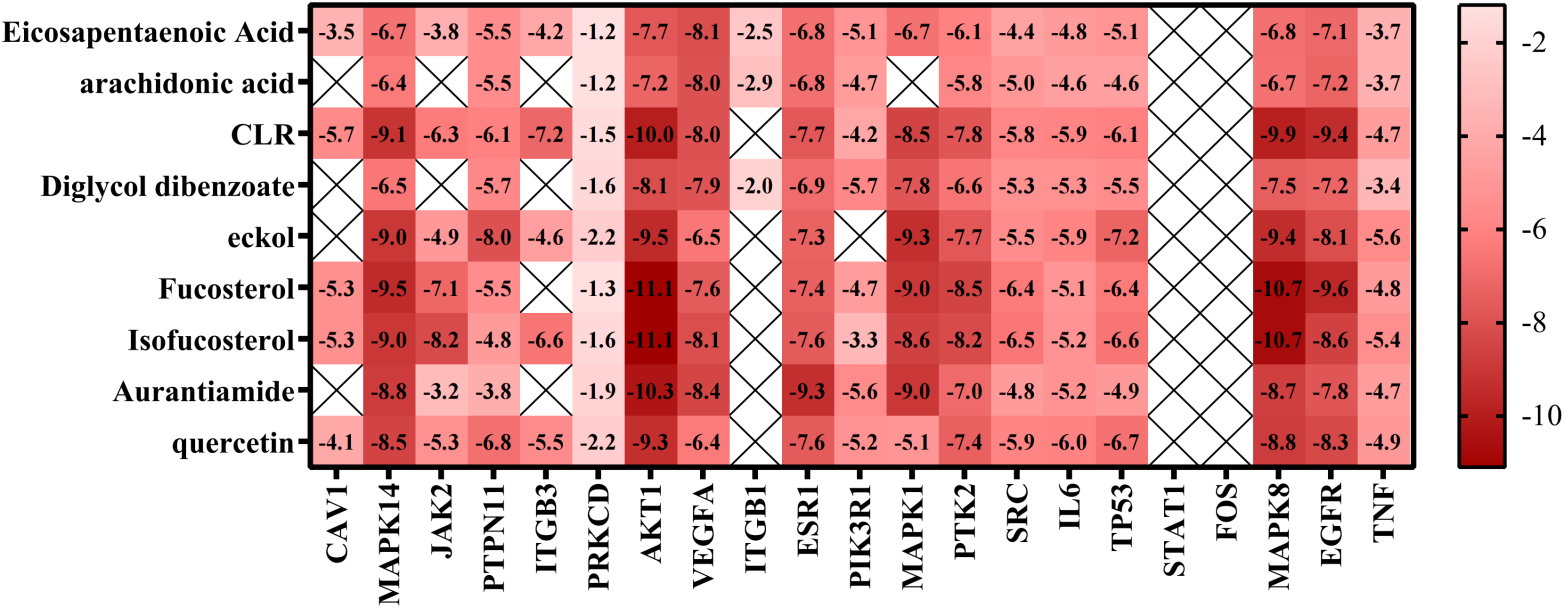
Thermographic analysis of molecular docking binding energy.

**Figure 7:**
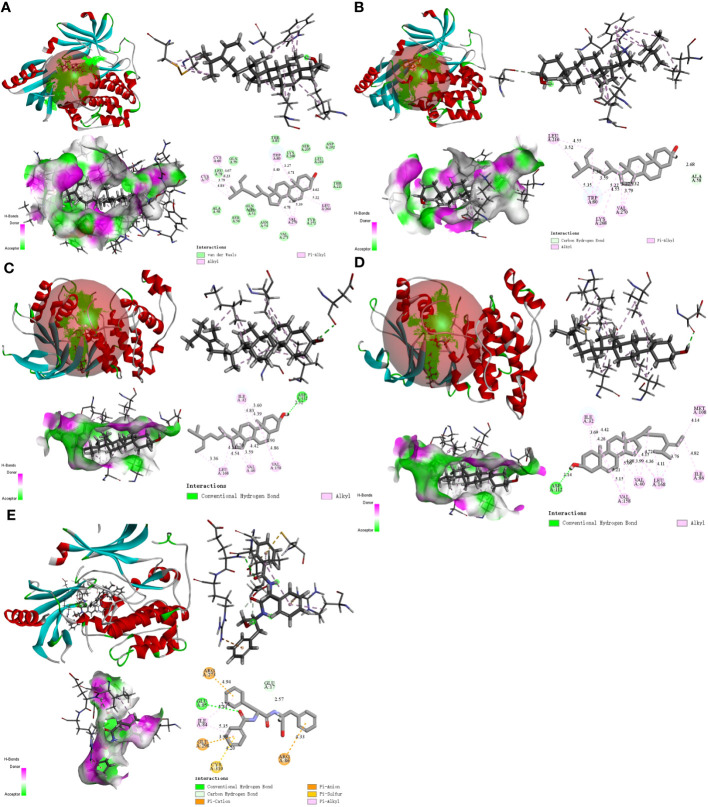
The 2D and 3D visualization plots of the top five compound–target dockings with the lowest values of docking scores. The **(A)** AKT1-Fucosterol, **(B)** AKT1-Isofucosterol, **(C)** MAPK8-Isofucosterol, **(D)** MAPK8-Fucosterol, **(E)** AKT1-Aurantiamide complexes.

We used molecular docking to identify chemicals from the PPI system based on GD-related targets. The relationships among essential active chemicals and crucial targets were examined using AutoDock4.2, Discovery Studio 2019 Client, and PyMOL software. Molecular structures of significant targets were obtained from RCSB PDB (**Table 2**). We did not find the PDB ID of FOS. Since the docking of CAV1, JAK2, and ITGB3 receptors to compounds was not successfully implemented in Autodock Vina, we redocked using the Genetic Algorithm in AutoDock 4.2., and the docking analysis yielded the binding scores (**Figure 6**). A lower value indicated a greater binding capacity. We discovered that van der Waals forces, hydrogen bonds, and aromatic stacking (Pi-Cation, Pi-Anion, Pi-Sulfur, Pi-alkyl, and alkyl interactions) were involved in the interactions between the active site residues of crucial targets and potential active substances. All active compounds exhibited an excellent binding affinity to specific core targets (only binding energies < -5 kcal/mol were demonstrated). Fucosterol-AKT1, Isofucosterol-AKT1, Isofucosterol-MAPK8, Fucosterol-MAPK8, and Aurantiamide-AKT1 were the top five binding modes (**Figure 7**). The Fucosterol-AKT1 complex was stabilized by 15 van der Waals forces and 5 Pi-alkyl and alkyl interactions with CYS 77, CYS 60, TRP 80, LEU 264, and VAL 270, respectively (**Figure 7A**). The Isofucosterol-AKT1 complex was stabilized by one hydrogen bond (1H-bond) with residue ALA 58, and four Pi-alkyl and alkyl interactions with LEU 210, TRP 80, LYS 268, and VAL 270 (**Figure 7B**). On the other hand, the Isofucosterol-MAPK8 complex was stabilized by 1H-bond with residue ASP 112 and four alkyl interactions with ILE 32, VAL 158, VAL 40, and LEU 168 (**Figure 7C**). Similarly, a 1H-bond with residue ASP 112 and six alkyl interactions with ILE 32, MET 108, VAL 158, VAL 40, LEU 168, and ILE 86 stabilized the Fucosterol-MAPK8 complex (**Figure 7D**). Finally, the Aurantiamide-AKT1 complex was stabilized by three Pi-cation and Pi-anion interactions with ARG 273, ARG 86, GLU 298, two H-bonds with residue GLU 85 and GLU 17, one Pi-alkyl bond with ILE 84, and one Pi-sulfur bond with CYS 310 (**Figure 7E**).

**Table 2:** Grid docking parameters in molecular docking.

Targets	PDB ID	UniProt ID	Center grid box		
			X center	Y center	Z center
CAV1	7SC0	Q03135	0	0	0
MAPK14	1OVE	Q16539	29.747	15.683	27.293
JAK2	4IVA	O60674	0.161	-11.654	-8.084
PTPN11	3B7O	Q06124	28.81	8.938	63.391
ITGB3	4G1M	P05106	-36.16	46.505	55.032
PRKCD	3UFF	Q05655	0	0	0
AKT1	4EJN	Q01314	30.889	52.185	19.493
VEGFA	4QAF	P15692	13.309	63.036	-0.942
ITGB1	7NXD	P05556	130.433	182.225	111.984
ESR1	5AAV	P03372	31.01	14.202	10.787
PIK3R1	3I5S	P27986	0	0	0
MAPK1	2OJG	P28482	-13.772	13.979	41.667
PTK2	1MP8	Q05397	36.299	-3.761	24.196
SRC	1O43	P12931	18.953	20.632	21.188
IL6	1ALU	P05231	-7.7	-12.7	0
TP53	4AGP	P04637	91.098	96.917	-46.275
STAT1	1YVL	P42224	-30.304	-13.959	146.805
MAPK8	4QTD	P45983	14.189	15.864	19.659
EGFR	5UG9	P00533	-8.371	17.712	-12.846
TNF	5UUI	P01375	41.438	43.125	1.22
FOS	–	P01100	–	–	–

### Molecular dynamics simulation of structural stability and interaction energy

**Figure 8:**
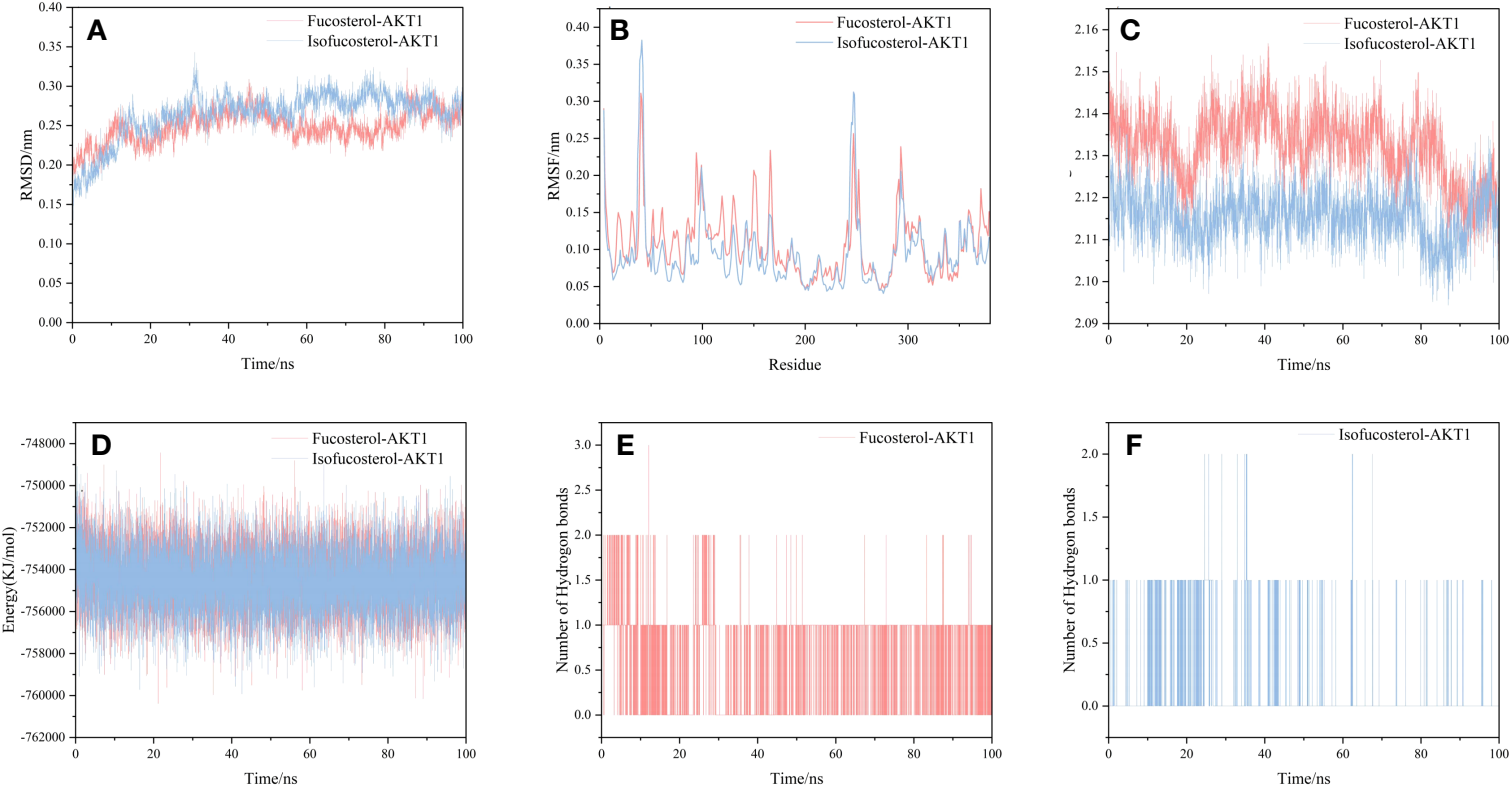
Results of molecular dynamics simulations. **(A)** The RMSD curves, **(B)** RMSF curves, **(C)** radius of rotation curves, **(D)** interaction energy curves, and number of hydrogen bonds for the **(E)** Fucosterol-AKT1 and **(F)** Isofucosterol-AKT1 complexes during the 100 ns simulations.

We selected and implemented the top two compound-target dockings (Fucosterol-AKT1 and Isofucosterol-AKT1) for MD simulations. After 100 ns of MD simulations, the dynamic variations of the Fucosterol-AKT1 and Isofucosterol-AKT1 complexes were assessed. Subsequently, we computed the Root-Mean-Square Deviation (RMSD) to understand the complexes’ molecular configurations and the system’s stability during simulation. The RMSD curve represents the location deviations in the protein. Fucosterol-AKT1 and Isofucosterol-AKT1 had average RMSD values of 2.5 and 2.6 Å, respectively. The RMSD of the Fucosterol-AKT1 complex was less than that of the Isofucosterol-AKT1 complex between the 45-85 ns time interval, and the RMSD curves relatively stabilized after 85 ns (**Figure 8A**). The Root-Mean-Square Fluctuation (RMSF) graph shows the protein amino acid residue variations. According to the findings, most simulations had small alterations in amino acid structure. Furthermore, most of the residues exhibited minor structural modifications. The RMSF values of residue numbers 50-200 in AKT1 after Fucosterol binding showed greater flexibility than the same regions in AKT1 after Isofucosterol binding (**Figure 8B**). The gyration (Rg) radius curve represents the tightness of the protein’s general configuration. The Fucosterol-AKT1 and the Isofucosterol-AKT1 complexes had stable rotation radii, although the former folded with greater force (**Figure 8C**). The binding free energy can be used to assess the change in the binding pattern and stability of ligands and proteins. Compared to the isofucosterol-AKT1 complex, which showed an average interaction energy and energy fluctuation of 144.536 kcal/mol and 17.43 kcal/mol, respectively, the fucosterol-AKT1 complex had an average interaction energy and energy fluctuation of 141.412 kcal/mol and13.63 kcal/mol, respectively (**Figure 8D**). The number of Hydrogen bonds (H-bonds) in a complex might reveal information about its binding strength. The ligands and residues of all five protein compartments created one or several hydrogen bonding connections. During the 100 ns simulations, the Fucosterol-AKT1 complex had a higher H-bond density and size (**Figures 8E, F**).

## Discussion

The GD etiology and pathogenesis in relation to heredity, mental stimulation, environment, infection, and other factors have not been fully elucidated. Severe GD cases might result in life-threatening complications such as liver damage, heart failure, and thyroid storm. Besides being ineffective, current GD therapies have also been linked with severe limitations, such as toxic pharmacological side effects, permanent hypothyroidism, vocal cord paralysis, and other destructive damages, that all cause clinical challenges. There has been a growing demand for novel strategies that utilize traditional approaches to treat GD. In this regard, as a complement, traditional medicine has been shown to generate synergistic results with minimal toxicity. Guided by TCM theory, HK was the most common drug combination used to treat GD. However, the molecular mechanisms of HK in GD treatment have not been fully explored. Herein, we searched and selected QFP compounds and targets from multiple databases and created a “compound-gene target-disease” network. The CytoNCA software was used to perform topological analysis to estimate key protein targets involved in GD treatment and 15 essential targets including AKT1, PIK3R1, MAPK1, MAPK8, MAPK14, VEGFA, EGFR, IL6, TNF, TP53, JAK2, STAT1, ESR1, FOS, and CAV1 were identified. Subsequently, we excluded a list of irrelevant pathways in the top 20 most enriched pathways to elucidate the pathogenesis of GD and establish a prospective network model. The PI3K-AKT and MAPK pathways were identified as the most prominent penetration points in GD treatment. Furthermore, the fundamental biological processes, molecular functions, and cellular components in GO enrichment analysis helped illustrate the multi-dimensional and multi-target therapeutic pathways. Finally, the computational and validation molecular docking and MD simulation processes enabled us to further discuss the biological function of HK in the GD treatment from a micro-level perspective and to present a theoretical framework for GD therapy with TCM.

In the TCM context, HK treats GD by softening firmness and dispersing stagnation. However, Haizao and Kunbu, known as iodine-rich Chinese medicine, have been controversial in GD treatment due to their higher iodine content. Wild Laminaria japonica (0.1-1.4 g) contains approximately 150 μg of iodine, while the iodine concentration of wild Sargassum is 58-629 μg/g (35). However, in China, HK refers to specially processed TCM pieces. According to current research, the iodine content range of Haizao and Kunbu pieces are 297.67-814.59 μg/g and 855.33-5481.33 μg/g, respectively (36, 37). Consistent with an Italian study, dried pieces had higher iodine content than fresh samples (38), which can also be attributed to the different plant origins. Here, we studied processed Chinese herbal pieces. Chinese herbal pieces should be decocted before use, and studies have shown that boiling in fresh water can reduce the seaweed iodine content (35). Additionally, following gastrointestinal digestion, only 49-82% of seaweed iodine appears to be available for human absorption (39). Despite its substantial iodine amounts, ingesting considerable quantities of HK does not necessarily indicate a risk of excessive iodine intake (40). There is a clinical belief that GD hyperthyroidism patients should strictly limit iodine intake. However, some new perspectives suggest that limiting iodine intake might not benefit GD patients in areas with adequate or excessive iodine intake (41). The considerable Chinese population that participated in the USI program for two decades has reached an iodine adequacy status, and the once-high hyperthyroidism prevalence has consequently been reduced to relatively stable levels (1). The latest study proves that the cumulative post-USI hyperthyroidism incidence in different iodine trophic status areas in China has not increased (42). Studies have also shown that limiting dietary iodine intake does not improve ATD effectiveness in GD treatment, nor does it increase the GD recurrence rate after ATD withdrawal, and even severe iodine intake restriction can negatively affect GD (43–45). Furthermore, some GD patients who received an acceptable excess of iodide during treatment experienced reduced hyperthyroidism and thiourea-related side effects (46). Moreover, this treatment did not affect the efficacy of radioactive iodine therapy (47). Furthermore, potassium iodide has been shown to be effective and safe in specific GD patients, such as pregnant and breastfeeding women, and patients with malignant tumors, or those undergoing radiation and chemotherapy (41, 48). An *in vivo* study from Japan recently suggested that the chronic anti-thyroid action of iodine in GD involves hormone secretion inhibition (49). In this context, researchers believe that iodine-rich TCMs such as HK can be rationally used in treating some GD patients, such as those who are intolerant to ATD or refuse surgical treatment, and patients with mild and moderate GD whose serum FT4 and Thyroid Stimulating Hormone (TSH) Receptor (TSHR) autoantibody (TRAb) levels are less than the upper limit of the detectable range in a laboratory (50–52). In our previous study, we demonstrated that an iodine-rich Chinese medicine formula could improve thyroid function and morphology in hyperthyroid rats (53). Furthermore, some previous clinical studies have proved that iodine-rich TCM for treating GD has the rapid onset, minor adverse reactions, and reduced serum TRAb advantages (54, 55). Some researchers in China have recently been conducting large-sample, multi-center, and strictly designed clinical studies on iodine-rich TCM for GD treatment (Registration numbers: ChiCTR2000032706, ChiCTR1900021572), and their findings will provide additional insights and inspiration for our subsequent research. Therefore, in future studies, we recommend a reasonable and comprehensive evaluation of the efficacy and safety of HK and further verification of its clinical effect in GD patients.

Based on our predictions, most HK active ingredients can influence biological processes, including proliferation, apoptosis, and inflammation. Aurantimide is a critical active small-molecule compound, and *in vitro* experiments have demonstrated that it could act on the PI3K/AKT signaling pathway (56) and exert anti-inflammatory effects via inhibiting the phosphorylation of the MAPK pathway (57). Eckol, a novel natural phizolian derived from marine brown algae, has been shown to downregulate EGFR, p-EGFR, JAK2, and STAT3 expression in tumor cells, indicating pro-apoptotic and anti-proliferative activities (58, 59). Eicosapentaenoic Acid (EPA), a polyunsaturated omega-3 fatty acid, has previously been reported to affect cell proliferation and inflammatory responses by inhibiting the phosphorylation of AKT (60, 61). Besides improving hormonal status in hyperthyroid rats, including T3 and TSH levels, EPA can also reduce the levels of pro-inflammatory cytokines, such as serum TNF-α (62). Fucosterol, an algae-derived unique plant sterol with various medicinal properties, has been predicted by studies to suppress the phosphorylation of Phosphatidylinositol 3-kinase/protein Kinase B (PI3K/Akt) signaling, reduce Mitogen-Activated Protein Kinase (MAPK) expression, and reduce IL-6 and TNF-α overexpression (63–65), influencing cell proliferation, apoptosis, and inflammation. On the other hand, quercetin is an excellent antioxidant that exerts sound anti-inflammatory effects and has been reported to inhibit the PI3k/Akt pathway by effectively binding to PIK3R1 (66, 67). Arachidonic acid metabolites are differentially affected by thyroid hormone status, and elevated levels of AA metabolites have been observed in the serum of patients with hyperthyroidism (68). We speculated that it is related to the Inhibitory effects of iodinated derivatives of arachidonic acid on iodine metabolism. Although the mechanism of the above active compounds in GD pathogenesis has not been fully explained, we could predict the optimal mechanism between compounds and target proteins by combining molecular docking results. Furthermore, verifying their biological feeatures in GD models is one of our future research directions.

The PPI network topology analysis revealed 21 core HK targets for treating GD. These protein targets are primarily involved in cell proliferation, apoptosis, and inflammatory processes in GD pathogenesis. The Vascular Endothelial Growth Factor (VEGF) family is required for the proliferation of blood vessels (69), and GD patients have elevated serum VEGF levels (70). According to research, iodide can decrease the expression of VEGFAs (VEGFs involved in promoting angiogenesis) upregulated by TSH (71). Besides eliminating vascular remodeling and inhibiting hair follicle hypertrophy (72), blocking VEGFA inhibits hyperthyroidism by increasing lymphatic flow in the Graves thyroid gland (73), decreasing thyroid weight during goiter development. Caveolin-1 (CAV1) is a member of the thyrosomal polyprotein complex required for thyroid hormone synthesis and thyroid cell homeostasis (74). Low CAV1 expression was observed in fat cells of GD patients (75). Pro-Epidermal Growth Factor (EGF) is an essential growth factor in thyroid tissue, and nuclear EGFR expression is elevated in GD tissue samples, implying that the EGFR-dependent modulation of thyroid cell proliferation under physiological conditions may be associated with hyperthyroidism (76). The Tumor Protein p53 (TP53) gene is important for inducing apoptosis or cell cycle interruption, and the succession of an insufficiently effective TP53 gene substantially raises the risk of developing GD. Given that the autoimmune thyroid illness may be accompanied by DNA damage and apoptosis, the insufficiently effective TP53 gene may initiate and sustain the autoimmune GD process (77). Furthermore, some well-known cytokines (IL6 and TNF-α) are an essential part of the autoimmune response in GD patients, which can promote inflammatory cell proliferation and infiltration into thyroid tissue and affect thyroid follicular cell growth and differentiation. Furthermore, these cytokines have been proven to be related to the recalcitrant nature of the disease and the severity of clinical symptoms (78–82). Other genes have also been found to be partially responsible for the regulated proliferation and Thyroid Hormone (TH) levels in GD (83–85). These gene targets imply that we can further investigate the mechanism of HK in GD treatment in terms of cell proliferation, apoptosis, and inflammatory processes.

**Figure 9:**
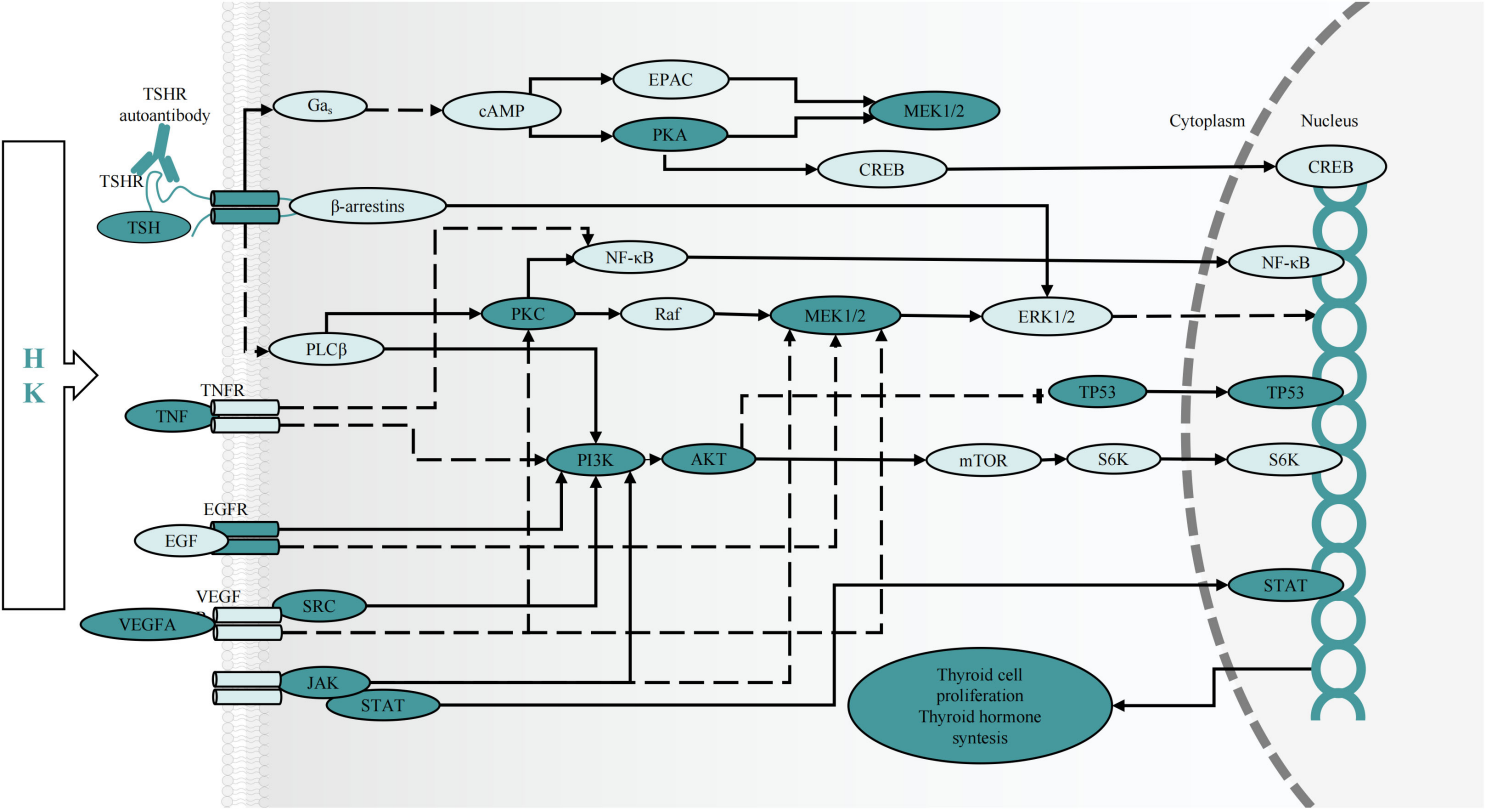
The predicted effect of HK on key targets and cascades in the treatment of GD. cAMP, cyclic adenosine monophosphate; CREB, cAMP response element-binding protein; EPAC, exchange protein activated by cAMP; MEK, mitogen-activated protein kinase kinase; mTOR, mammalian target of rapamycin; NF-кB, nuclear factor-кB; PLC, phospholipase C; Raf, RAF proto-oncogene serine/threonine protein kinase; PKA, protein kinase A; PKC, protein kinase C; S6K, ribosomal protein S6 kinase 1.

Additionally, KEGG enrichment analysis demonstrated that the PI3K-AKT and MAPK signaling pathways were involved in GD onset. Furthermore, some of the BP and MF items examined by GO, including protein phosphorylation, positive cell death regulation, cell activation, kinase activity regulation, MAPK cascade regulation, cell population proliferation, Transcription Factor (TF) binding, protein serine/threonine kinase activity, cytokine receptor binding, and Cell Adhesion Molecule (CAM) binding, revealed the significance of cell proliferation, differentiation, protein phosphorylation, and protein kinases in GD pathogenesis. Notably, GD patients have thicker, hypertrophied follicular cells in their thyroid glands that produce active thyroglobulin. The gland exhibits classic lymphocytic infiltrates considered to be principally connected with TSHR autoantibody secretion. Additionally, a histological examination showed occasional apoptotic cells and partial follicular wreckage (86). The presence of immune inflammation, cell proliferation, and apoptosis were all essential components in the pathological mechanism of GD. Although studies have reported that GD patients have all three types of TSHR autoantibodies, stimulating antibodies constitute the distinguishing characteristic of Grave’s hyperthyroidism. When stimulated, TSHR autoantibodies can induce complex signaling cascades, mainly activating GΑs and inducing cAMP/PKA pathways. The production of cAMP activates the cAMP Response Element-Binding Protein (CREB) and protein kinase A, which are directly or indirectly involved in inflammatory mediator generation, thyroid hormone synthesis, and thyroid cell proliferation (4, 87). The bioactive exertion of cAMP partially depends on the phosphorylation of the MAPK and PI3K/AKT pathways (88). Additionally, the binding of Β-arrestin attracted by TSHR to the receptor can activate the MAPK pathway (89), inducing protein synthesis, cell differentiation, and angiogenesis via hemodynamic effects (4, 90). Protein Kinase B (PKB), also known as AKT, is an intracellular signaling pathway phosphorylated and activated on the plasma membrane. Once activated, AKT regulates cell survival/apoptosis, cell proliferation, and protein synthesis. Recent findings have confirmed the involvement of the PI3K-AKT pathway in GD pathogenesis (91–93). Phosphorylated AKT stimulates the activation of TFs CREB and NF-kB, as well as inflammatory gene expression (such as IL-6) (94). Previous research showed that the cAMP/PKA pathway increased IL-6 production in thyroid cells via processes influencing the stability of IL-6 mRNA, IL-6 gene promoter, and c-Fos expression (95). Increased inflammatory chemical levels further worsen thyroid follicular cell stimulation and destruction, causing the secretion of more thyroid hormones and amplifying the body’s inflammatory response (96). Moreover, Janus Kinase (JAK)/Signal Transducer and Activator of Transcription (STAT) and their downstream effectors are the primary signaling cascades in TSHR activation. Our results and previous findings suggest that JAK2 and STAT1 phosphorylation may occur during GD treatment (97, 98). All the above-mentioned cascades have relevance to cellular development, survival, differentiation, cytokine and chemokine release, and apoptosis induction (99), indicating that thyroid cell activation and proliferation can be partially influenced via the regulation of protein phosphorylation within these central signaling cascades by HK (**Figure 9**).

MD simulation is an effective technique for examining molecular mechanisms, especially in evaluating the binding stability (100) and the selectivity of specific ligands for their target proteins (101). In this study, we conducted MD simulations to gain deeper insights into the stability of the protein-ligand complexes, specifically focusing on the Fucosterol-AKT1 and Isofucosterol-AKT1 complexes. We assessed the RMSD profiles of these complexes, which exhibited consistent patterns with RMSD values hovering around 0.27 nm. This pattern suggests that the system reached equilibrium during the simulation. We extended the MD simulation duration for these two complexes to generate a stable RMSD profile. In addition to RMSD, we evaluated several other factors, including RMSF, Rg, H-bonds, and binding free energy. These assessments provided further insights into the dynamic properties of the protein-ligand interactions. Overall, these results suggest that during MD simulation, fucosterol-AKT1 and isofucosterol-AKT1 complexes were stable and equilibrated.

Although network pharmacology, molecular docking, and molecular dynamics simulation methods were implemented to characterize the potential chemicals and targets of HK, there are some several limitations that need to be acknowledged. First, to accurately comprehend the behavior of chemical elements functioning on disease targets, network pharmacology can be utilized to predict the up-regulation and down-regulation of targets. Second, *in-vivo* and *in-vitvo* experiments should be conducted to accurately explore the therapeutic effects and associated mechanisms of the potential compound and target pairs. It is also necessary to identify the active ingredients with therapeutic effects in HK through basic experiments. These will be the focus of our future research.

## Conclusion

GD is an autoimmune disease caused by genetic and environmental factors. The multifaceted nature of GD calls for a multifaceted approach to its treatment, often involving multiple targets. There is a growing interest in innovative treatment strategies that draw from the wisdom of traditional medicine and apply it to the clinical management of GD. These strategies have demonstrated favorable efficacy and safety profiles. Nine compounds including Fucosterol, Isofucosterol, Aurantiamide, Eicosapentaenoic Acid, quercetin and eckol of HK were combined to 21 core targets including AKT1, PIK3R1, MAPK1, MAPK8, MAPK14, VEGFA, EGFR, IL6, TNF, TP53, CAV1, JAK2, and STAT1, and by participating in biological processes such as cell population proliferation, protein phosphorylation, regulation of kinase activity and protein serine/threonine kinase activity and PI3K-AKT, MAPK, and other pathways play a crucial role in the treatment of GD. Molecular docking and molecular dynamics simulation demonstrated that the Fucosterol-AKT1 and Isofucosterol-AKT1 complexes exhibited the highest binding energy, indicating that HK contains key compounds and targets. While future research will necessitate further biological experiments, including additional *in vitro* and *in vivo* studies, our current findings suggest that HK holds significant promise as an effective herbal remedy for treating GD.

## Data availability statement

The original contributions presented in the study are included in the article/**Supplementary Materials**, further inquiries can be directed to the corresponding author/s.

## Author contributions

Methodology: MY, YL, XH. Software: MY, XH. Validation: MY, XH. Formal analysis: MY, XH. Visualization: MY, YL, QL, YA. Conceptualization: YL, TG. Investigation: YL, DG, QL, YW, YA. Supervision: YL, TG. Resources: DG, QL, YW, YA. Data Curation: DG, QL, YW. Writing - Original Draft: MY. Writing - Review & Editing: YL, XH, TG. Project administration: YL, TG. Funding acquisition: YL. All authors contributed to the article and approved the submitted version.
